# Synthetic Approaches for Poly(Phenylene) Block Copolymers via Nickel Coupling Reaction for Fuel Cell Applications

**DOI:** 10.3390/polym12071614

**Published:** 2020-07-20

**Authors:** Adam F. Nugraha, Songmi Kim, Farid Wijaya, Byungchan Bae, Dongwon Shin

**Affiliations:** 1 Fuel Cell Laboratory, Korea Institute of Energy Research, Daejeon 34129, Korea; adamfn@kier.re.kr (A.F.N.); songmk307@kier.re.kr (S.K.); faridwijaya@ust.ac.kr (F.W.); bcbae@kier.re.kr (B.B.); 2 Department of Renewable Energy Engineering, University of Science and Technology (UST), Daejeon 34113, Korea; 3 Department of Chemical Engineering, Yonsei University, Seoul 03722, Korea

**Keywords:** poly(phenylene), multi-block copolymer, nickel coupling reaction, anion-exchange membranes, ion conductivity

## Abstract

Several methods to synthesize poly(phenylene) block copolymers through the nickel coupling reaction were attempted to reduce the use of expensive nickel catalysts in polymerization. The model reaction for poly(phenylene) having different types of dichlorobenzene derivative monomers illustrated the potential use of cost-effective catalysts, such as NiBr_2_ and NiCl_2_, as alternatives to more expensive catalysts (e.g., bis(1,5-cyclooctadiene)nickel(0) (Ni(COD)_2_)). By catalyzing the polymerization of multi-block poly(phenylene) with NiBr_2_ and NiCl_2_, random copolymers with similar molecular weights could be prepared. However, these catalysts did not result in a high-molecular-weight polymer, limiting their wide scale application. Further, the amount of Ni(COD)_2_ could be reduced in this study by approximately 50% to synthesize poly(phenylene) multi-block copolymers, representing significant cost savings. Gel permeation chromatography and nuclear magnetic resonance results showed that the degree of polymerization and ion exchange capacity of the copolymers were almost the same as those achieved through conventional polymerization using 2.5 times as much Ni(COD)_2_. The flexible quaternized membrane showed higher chloride ion conductivity than commercial Fumatech membranes with comparable water uptake and promising chemical stability.

## 1. Introduction 

Polymer electrolyte membrane fuel cells (PEMFCs) have received considerable attention as efficient and environmentally friendly energy devices [1,2]. PEMFCs are known to be suitable for both stationary and portable applications, including electric vehicles, but they require an expensive platinum catalyst because of the slow oxygen reduction reaction under the acidic operating conditions [3,4,5]. As an alternative, alkaline membrane fuel cells (AMFCs) seem to be promising because they involve an electrochemical reaction under basic operating conditions, which enables the use of more abundant non-noble-metal catalysts [6,7,8,9]. Although AMFCs are economically more advantageous than PEMFCs, the search for a suitable, highly ion-conductive, chemically stable electrolyte membrane has been a challenge for polymer scientists for the last few decades [10].

Since the use of Nafion was first reported for PEMFCs, no similar benchmark membranes have been developed for AMFC applications. Although a few membranes are available from Fumatech and Tokuyama, these membranes exhibit low conductivity and stability. Thus, highly conductive and chemically stable anion-exchange membranes (AEMs) have been extensively developed by modifying conventional polymer backbones such as polyethylene, polystyrene, and poly(ether sulfone)s copolymers [11,12,13,14,15,16,17]. In addition, numerous synthetic routes for introducing various anion-exchange functional groups have been proposed [9,18,19,20]. For example, halogenation processes such as chloromethylation and bromination have been widely used to introduce anion-exchange groups into the polymer backbone, followed by a quaternization process [21,22]. However, this process was found to induce side reactions such as crosslinking [20].

Among various polymer backbones, quaternized aromatic polymers are preferred to be used as polymer electrolytes for AMFCs because of their excellent mechanical properties, thermal stability, and favorable hydroxide conductivity. Commonly used polyaromatic electrolytes include quaternized poly(arylene oxide)s, poly(aryl ether ketone)s, and poly(aryl ether sulfone)s, all of which are synthesized via polycondensation reactions [18,23,24,25,26]. The anion conductivity of quaternized aromatic polymers has been reported to increase with the increasing water content, as also observed in proton exchange membranes (PEMs), as both membranes undergo a similar water-assisted ion transport mechanism [27,28]. Therefore, controlling the polymer morphology, which is a critical factor for ion transport, is essential to maximizing the utilization of water molecules to achieve high ion conductivity. Thus, introducing a multi-block polymer backbone may be a promising approach for enhancing the performance of hydrophilic–hydrophobic phase-separated membranes [9,29,30,31]. 

For the long-term stability of AMFCs, the chemical instability of AEMs has also been a formidable concern, particularly because the aryl-ether group (C–O–C bonds) in the aromatic polymers is chemically unstable under alkaline conditions [21,22,32,33]. The ether linkages close to electron-withdrawing groups such as benzyl trimethylammonium are not resistant to hydroxide ion attack owing to the simultaneous electron deficiency of the aryl group and the low dissociation energy of the ether substituent, thus leading to polymer backbone degradation [21,22,32,33]. Therefore, to chemically stabilize AEMs under harsh alkaline conditions, a few research groups have attempted to prepare an aryl-ether-free polyaromatic electrolyte membrane [34,35]. The introduction of a poly(phenylene) backbone can be considered a promising approach for improving AEM stability because of its extremely stable chemical structure.

The nickel-catalyzed carbon–carbon coupling reaction has been used for decades as a method for successfully synthesizing a poly(phenylene) backbone [36,37,38]. Although the Ni(0) coupling reaction seems to be the only promising synthetic route for obtaining high-molecular-weight poly(phenylene)s, Ni(0) catalysts are typically sensitive to oxidation. Bis(1,5-cyclooctadiene)nickel(0) (Ni(COD)_2_) has been extensively used as the main precatalyst for various applications owing to its stability under an inert atmosphere and low temperatures [10]. However, despite its favorable reactivity, it is limited by its complicated setup, such as the Schlenk technique or the use of a glove box to avoid immediate decomposition upon exposure to air. Hence, the development of alternative Ni(0)-olefin precatalyst, which is stable in air and has reactivity comparable with that of Ni(COD)_2_, has been a main research goal for many years. Very recently, a new Ni(0)-olefin precatalyst was reported that exhibited similar reactivity with Ni(COD)_2_ [39]. Although this Ni(0)-olefin complex, which has *p*-CF_3_-stilbene derivatives as ligands, offers the advantages of being robust, air-stable, and easy to handle in open flask conditions, its commercialization requires more relevant studies. The cost-effective polymerization of poly(phenylene)s requires either a lower amount of expensive Ni(COD)_2_ or a process that involves the use of low-cost catalysts such as NiBr_2_ and NiCl_2_.

In this study, we investigated the viability of decreasing the amount of expensive Ni(COD)_2_ catalysts as well as the applicability of low-cost Ni catalysts by adopting a model reaction for random and block copolymer polymerizations. First, we investigated the feasibility of two other nickel catalysts, NiBr_2_ and NiCl_2_, for a model reaction using 2,5-dichloro*-p-*xylene (2,5-DCPX) and 2,4-dichlorotoluene (2,4-DCT) as starting materials. Next, we used these catalysts to synthesize a model multi-block polymer from chlorine-telechelic aromatic oligomers and 3,5-dichlorobenzophenone (3,5-DCBP) monomers. Although NiBr_2_ and NiCl_2_ were effective in synthesizing the random copolymers, only the Ni(COD)_2_ catalyst provided a high molecular weight for the model multi-block polymer. Finally, we focused on decreasing the amount of Ni(COD)_2_ for the synthesis of the amine-containing multi-block poly(phenylene) and successfully decreased the catalyst amount to 50% of the reported value for the polymerization. The model reaction and polymerization were characterized in detail by gel permeation chromatography (GPC) and nuclear magnetic resonance (NMR). The multi-block poly(phenylene) produced by the optimized synthesis was cast into AEMs by solution casting followed by a quaternization process. Then, the water uptake, ion conductivity, and long-term chemical stability of these membranes were tested for application in AMFCs.

## 2. Materials and Methods 

### 2.1. Materials

3,5-Dichlorobenzoyl chloride (3,5-DCBC, ≥95%), lithium aluminum hydride (LAH, >95%), 4,4′-dichlorodiphenyl sulfone (>98%), 2,2-bis(4-hydroxyphenyl)hexafluoropropane (>98%), *m*-xylene (>99.0%), and 2,2′-bipyridyl (BPY, >99%) were purchased from Tokyo Chemical Industry (Tokyo, Japan). Dichloromethane (DCM, anhydrous, ≥99.8%), triethylamine (≥99.5%), dimethylamine hydrochloride (99%), 1-methyl-2-pyrrolidinone (NMP, anhydrous, ≥99.9%), Ni(COD)_2_, nickel(II) bromide (NiBr_2_, 98%), nickel(II) chloride (NiCl_2_, 98%), zinc (dust, <10 μm, ≥98%), 2,5-DCPX (97%), 2,4-DCT (98%), benzene (anhydrous, 99.8%), triphenylphosphine (PPh_3_, ReagentPlus^®^, 99%), aluminum chloride (AlCl_3_, ReagentPlus^®^, 99%)**,** potassium carbonate (anhydrous, free-flowing, Redi-Dri™, ACS reagent, ≥99%), iodomethane (containing copper as stabilizer, ReagentPlus^®^, 99%), lithium bromide (LiBr, reagent plus ≥99%), chloroform-*d_1_* (CDCl_3_; 99.8 at% D, containing 0.1 *v*/*v* % tetramethylsilane (TMS)), and dimethyl sulfoxide-*d_6_* (DMSO-*d_6_*, 99.9 at% D) were purchased from Sigma Aldrich (St. Louis, MO, USA). Toluene (extra pure), *n*-hexane (extra pure), hydrochloric acid (extra pure), sodium bicarbonate, ethyl ether (extra pure), DMSO (extra pure), and dimethylacetamide (DMAc, extra pure) were purchased from Duksan Pure Chemicals (Ansan, Korea). Methanol and ethanol were purchased form Samchun Pure Chemical (Pyeongtaek, Korea). The FAA-3-50 membrane was purchased from Fumatech (St. Ingbert, Germany). Potassium carbonate was dried at 160 °C overnight before use. Zinc dust was treated with 1 M HCl in water for 10 min and then washed with ethyl ether several times and dried under vacuum at 120 °C overnight. NiBr_2_ and NiCl_2_ were dried at 120 °C under vacuum overnight. BPY and PPh_3_ were stored in a glove box under an argon atmosphere before use. All other materials were used as received.

### 2.2. Syntheses of Random Poly(Phenylene)s

**Scheme 1:**
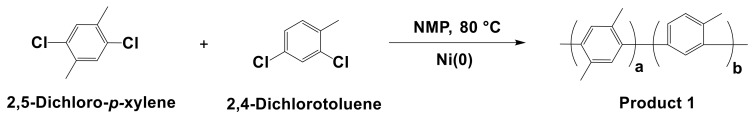
Synthesis of Product 1 as a model reaction.

**Scheme 2:**
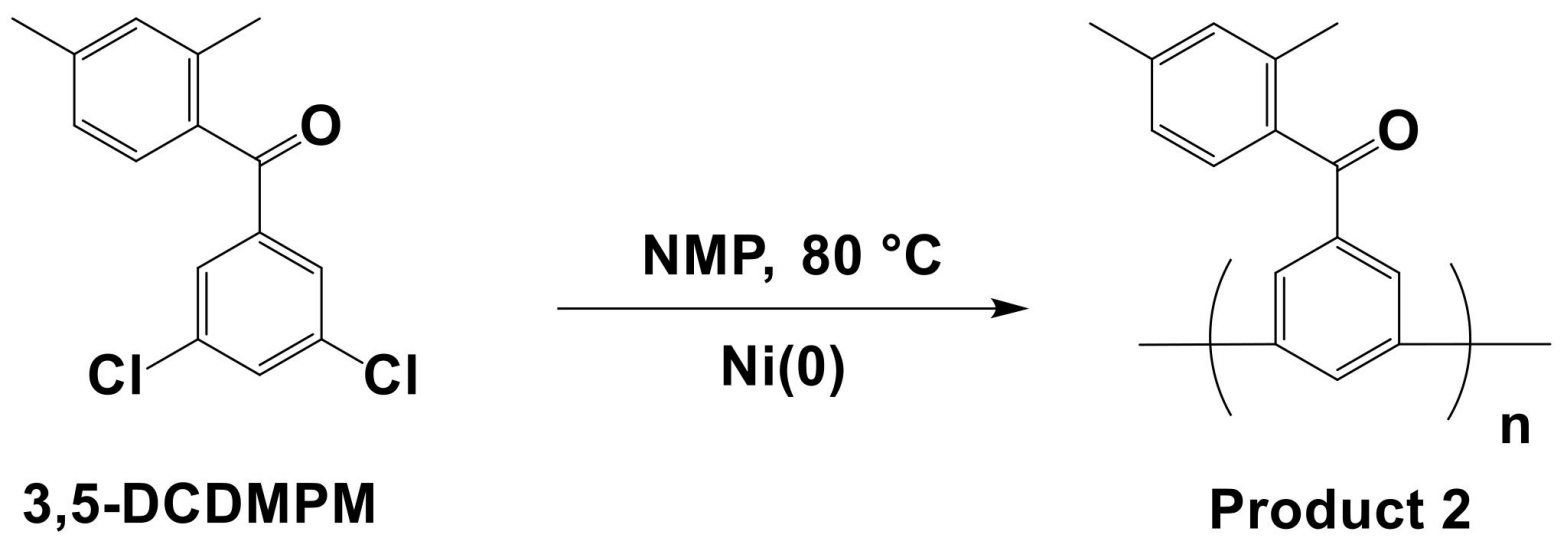
Synthesis of Product 2 as a model reaction.

As model reactions, random poly(phenylenes) were synthesized using Ni(COD)_2_, NiBr_2_, and NiCl_2_ to obtain Product 1 (Scheme 1) and Product 2 (Scheme 2). The typical synthetic procedure for the model reaction using Ni(COD)_2_ is as follows: 2,5-DCPX (0.25 g, 1.4 mmol), 2,4-DCT (0.23 g, 1.4 mmol), and BPY (1.14 g, 7.2 mmol) were dissolved in 5 mL of NMP and mixed in a reactor equipped with a mechanical stirrer at 80 °C under an argon purge. Next, Ni(COD)_2_ (2.0 g, 7.2 mmol) was added as a catalyst, and the reaction conditions were maintained for 4 h. After the reaction, the solution was precipitated into 6 M HCl in methanol and then rinsed with methanol several times. The white precipitate of Product 1 was filtered and dried under vacuum at 80 °C overnight; the molecular weight was estimated by GPC.

To use the NiX_2_ as the catalyst, either NiBr_2_ (0.11 g, 0.5 mmol) or NiCl_2_ (0.07 g, 0.5 mmol), zinc (1.78 g, 27.5 mmol), PPh_3_ (0.64 g, 3.2 mmol), and 5 mL of NMP were charged into a reactor equipped with a mechanical stirrer under an argon purge. After the solution stabilized at 80 °C, a solution of 2,5-DCPX (0.56 g, 3.2 mmol) and 2,4-DCT (0.5 g, 3.2 mmol) in 5 mL of NMP was added by injection, and the reaction was maintained for 24 h. After the reaction, the solution was precipitated into 6 M HCl in methanol and then rinsed with methanol several times. The precipitate was then filtered and dried under vacuum at 80 °C overnight; the molecular weight was estimated by GPC (Younglin Instruments, Anyang, Korea). Product 2 was synthesized by using (3,5-dichlorophenyl)-(2,4-dimethylphenyl)methanone (3,5-DCDMPM) as the monomer following the same procedure as that for Product 1 with each catalyst.

### 2.3. Syntheses of Model Poly(Phenylene) Multi-Block Copolymer with NiX_2_ and Ni(COD)_2_

**Scheme 3:**
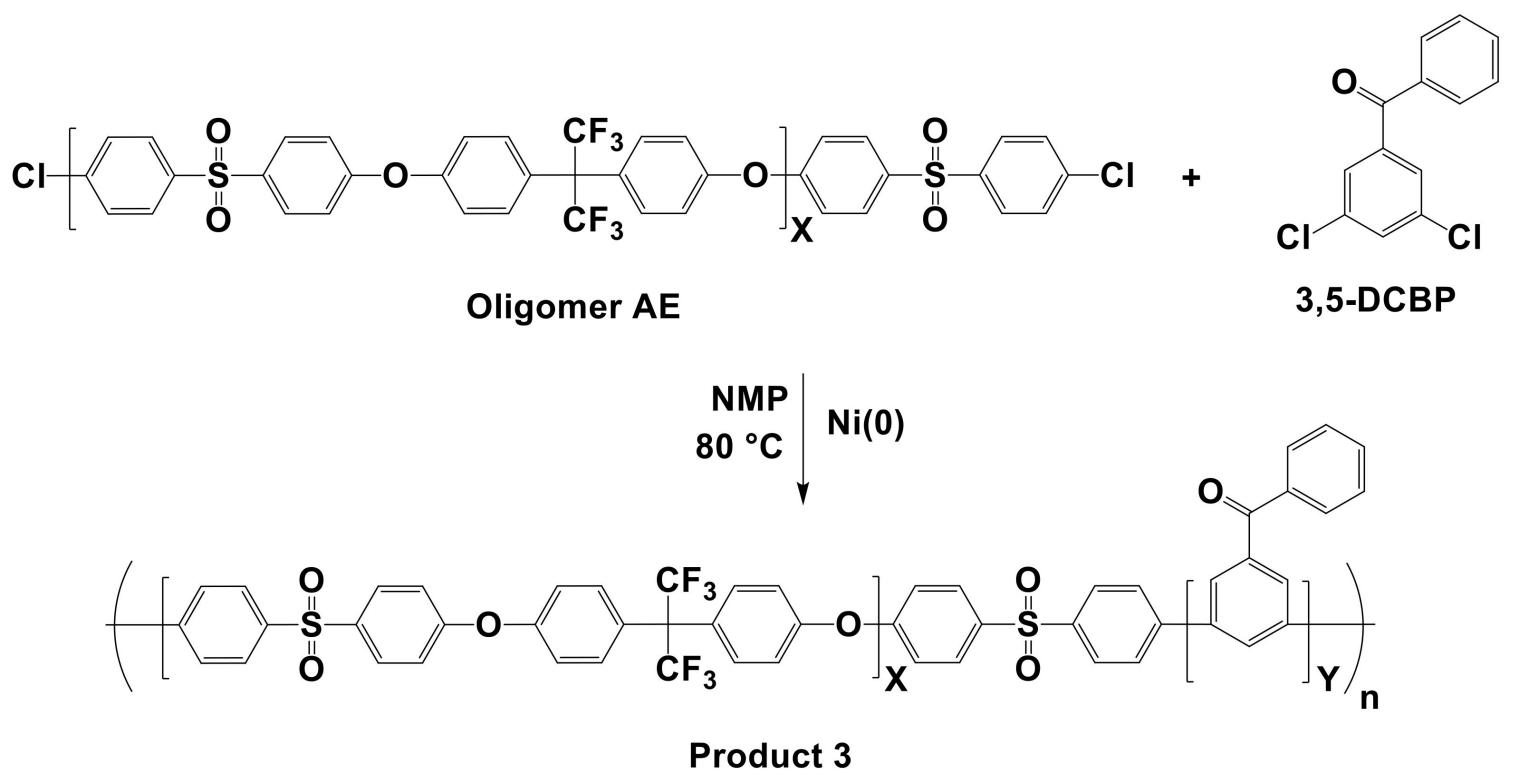
Synthesis of Product 3 using NiBr_2_, NiCl_2_, or Ni(COD)_2_ as catalysts.

The syntheses of model poly(phenylene) block copolymers (Product 3) were catalyzed by Ni(COD)_2_, NiBr_2_, and NiCl_2_, as shown in Scheme 3. The typical procedure for Product 3 using Ni(COD)_2_ is almost the same as that for Product 1. 2,5-DCPX and 2,4-DCT were replaced with a chlorine-end-capped aryl ether oligomer (Oligomer AE) and 3,5-DCBP, respectively, to synthesize model multi-block copolymers. The polymerization reaction was maintained for 4 h, and the solution was precipitated into 6 M HCl in methanol followed by rinsing with methanol several times. The precipitate was filtered and dried under vacuum at 80 °C overnight to obtain the product, and the molecular weight was estimated by GPC. Polymerization with NiBr_2_ and NiCl_2_ followed the same procedure as that for Product 1. After the polymerization, the purification step was identical to that used for Products 1 and 2.

### 2.4. Syntheses of the Amine-Containing Multi-Block Copolymer with Ni(COD)_2_

**Scheme 4:**
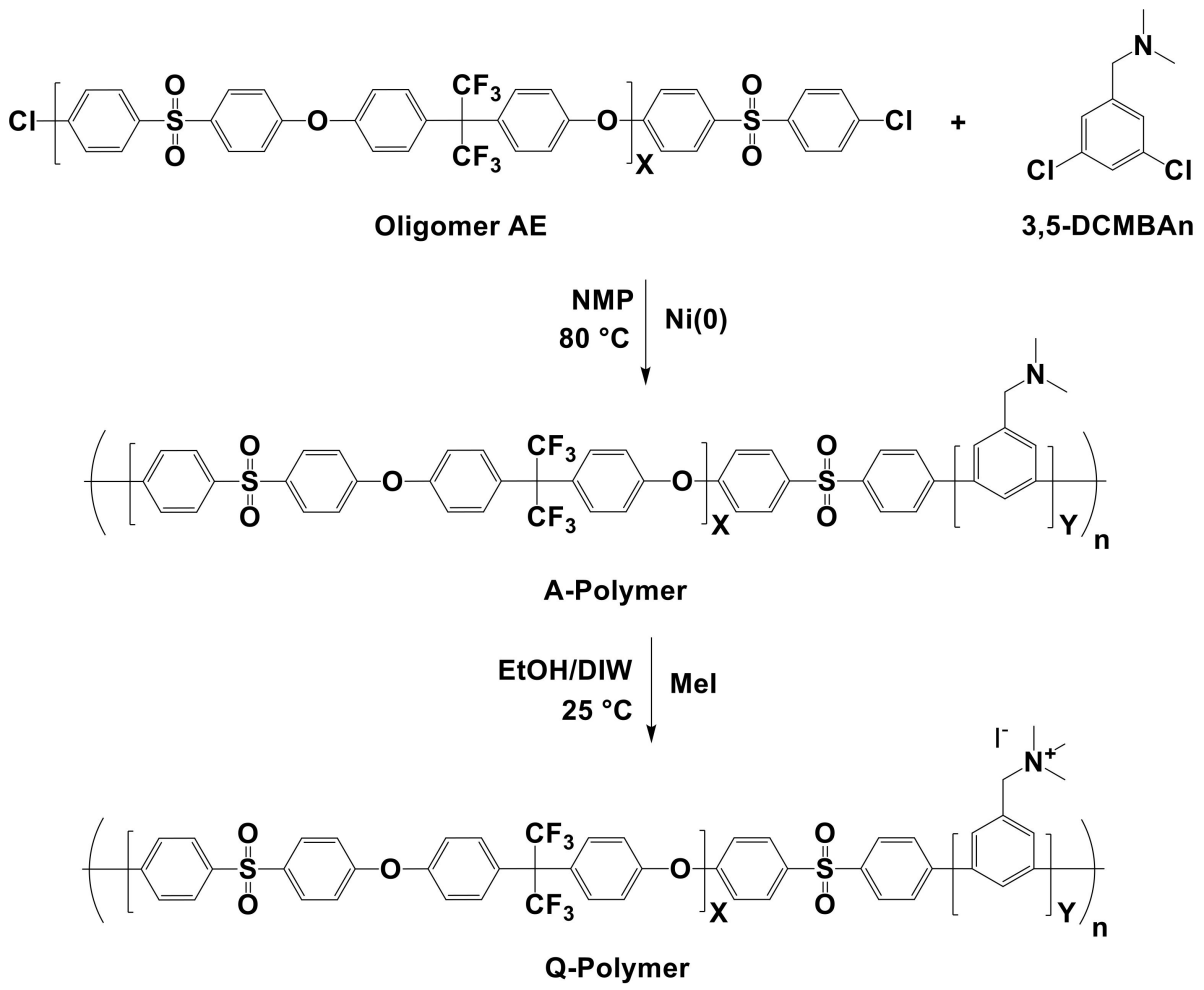
Polymer synthesis with Ni(COD)_2_.

The amine-containing multi-block copolymers (A-Polymer) were synthesized using Ni(COD)_2_, as shown in Scheme 4. The typical procedure is similar to the polymerization of Product 3. Oligomer AE was introduced as the hydrophobic component, and 3,5-DCBP was replaced by 3,5-dichloro-*N,N*-dimethylbenzylamine (3,5-DCMBAn) as the precursor hydrophilic component. The feeding ratio of Oligomer AE and 3,5-DCMBAn was maintained at 1:9 to obtain a membrane with 1.80 mEq g⁻^1^. During the purification step, the obtained product was washed with a saturated sodium hydrogen bicarbonate aqueous solution, deionized water (DIW), and methanol several times. The polymer was vacuum-dried at 70 °C (2.24 g, 84.7% yield).

### 2.5. Membrane Preparation and Quaternization

The A-Polymer (0.6 g) was dissolved in NMP (6 mL), and the solution was cast onto a flat glass plate and dried overnight on a hot plate at 70 °C in air. The obtained film was further dried in a vacuum oven at 80 °C for 6 h to produce a tough, transparent membrane with a thickness of 30 ± 3 µm. The membrane was immersed in 5 *v*/*v*% iodomethane in an ethanol/DIW (2:1) solution for three days to obtain a quaternized membrane in an iodide ion form. Then, the membrane was separately treated with 1 M NaOH and 1 M NaCl aqueous solutions for 24 h each, followed by washing with DIW several times and drying at room temperature to obtain the membrane in a chloride ion form.

### 2.6. Characterization

The proton ^1^H NMR spectra were recorded on an AVANCE III (600 MHz) spectrometer (Bruker BioSpin, Billerica, Massachusetts, US), using DMSO-*d_6_* or CDCl_3_ as solvents and 0.1 *v*/*v* % TMS as an internal reference. The molecular weights of the polymers were estimated by a GPC instrument equipped with a Yonglin YL9112 isocratic pump and an YL 9120 UV–visible detector (Younglin Instruments, Anyang, Korea). Shodex KF-805L and SB-803HQ columns (Showa Denko K. K., Tokyo, Japan) were used to analyze polymers. DMAc containing 0.01 M LiBr was used as the eluent. Molecular weights were calibrated using polystyrene standard samples.

Membrane ion conductivity (σ) was measured using a four-electrode conductivity cell (WonATech, Seoul, Korea) equipped with a Solartron 1260 impedance/gain-phase analyzer and a Solartron 1287 electrochemical interface (Solatron, Farnborough, UK) and was calculated as

(1) $$\sigma =\frac{D}{L\times T\times R},$$

where *D* is the distance between the electrodes, *L* is the membrane width, *T* is the membrane thickness, and *R* is the impedance resistance obtained from the impedance plot in the frequency range of 10^−1^ to 10^5^ Hz. Chloride ion conductivity was measured in 25 °C DIW. 

The water uptake was measured by preparing 2 × 2 cm^2^ samples, which were dried under vacuum at 100 °C for 24 h and immediately weighed on an analytical balance (EM 120-HR, Precisa, Dietikon, Switzerland) at 100 °C. The samples were then soaked in DIW at room temperature for 24 h. The sample weight was measured after removing excess water on the surface. The water uptake was calculated as follows

(2) $$\mathrm{Water}\;\mathrm{uptake}\;(\%)=\frac{\left(W_{w}\;-\;W_{d}\right)}{W_{d}}\;\times \;100,$$

where *W_d_* and *W_w_* are the weights of the dry and fully hydrated membrane, respectively.

The densities of the dried Q-Polymer and Fumatech membranes were assumed to be ~1.5 g/cm^3^ based on a previous report [40]. The water volume fraction was calculated from the density and water uptake data by using the following equation

(3) $$\varphi \left(H_{2}O\right)=\frac{\rho_{H_{2}O}}{\left(\frac{M_{H_{2}O}}{\rho_{H_{2}O}}+\frac{1}{\rho_{polymer}}\times \frac{100}{W\left(\%\right)}\right)},$$

where *ρ_H_2_O_* is the density of water at 80 °C, *M_H_2_O_* is the molecular weight, and *W*(%) is the water uptake.

The long-term chemical stability was tested by immersing each membrane equipped with WonATech four-electrode conductivity cells in 1 M aqueous NaOH at 25 °C for 700 h under a nitrogen purge. The NaOH solution was renewed every three days to prevent contamination with CO_2_. At the same time, the membrane was washed with DIW several times, and its ion conductivity was measured via impedance spectroscopy in DIW at 25 °C.

## 3. Results and Discussion

### 3.1. Synthesis of Random Poly(Phenylene)s

The model reaction for synthesizing random poly(phenylene)s was performed to investigate the performance of three different Ni catalysts. Product 1 was synthesized from commercially available 2,5-DCPX and 2,4-DCT in the presence of NMP and the nickel catalyst, as shown in Scheme 1. Ni(COD)_2_, NiBr_2_, and NiCl_2_ were selected as the nickel catalysts for the polymerization, which was monitored by GPC and NMR.

**Scheme 5:**
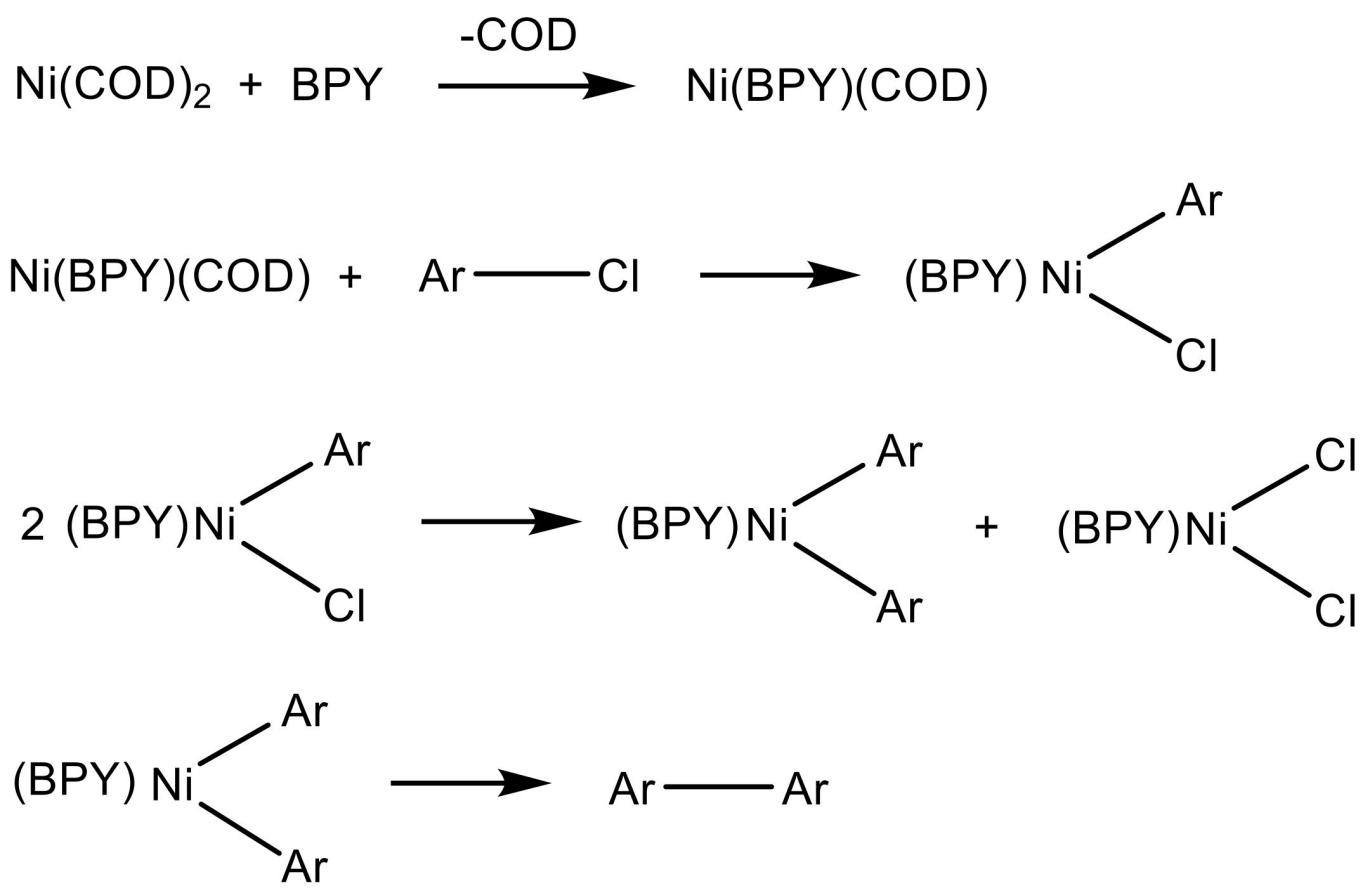
Mechanism of the nickel coupling reaction with bis(1,5-cyclooctadiene)nickel(0) (Ni(COD)_2_) [41].

**Scheme 6:**
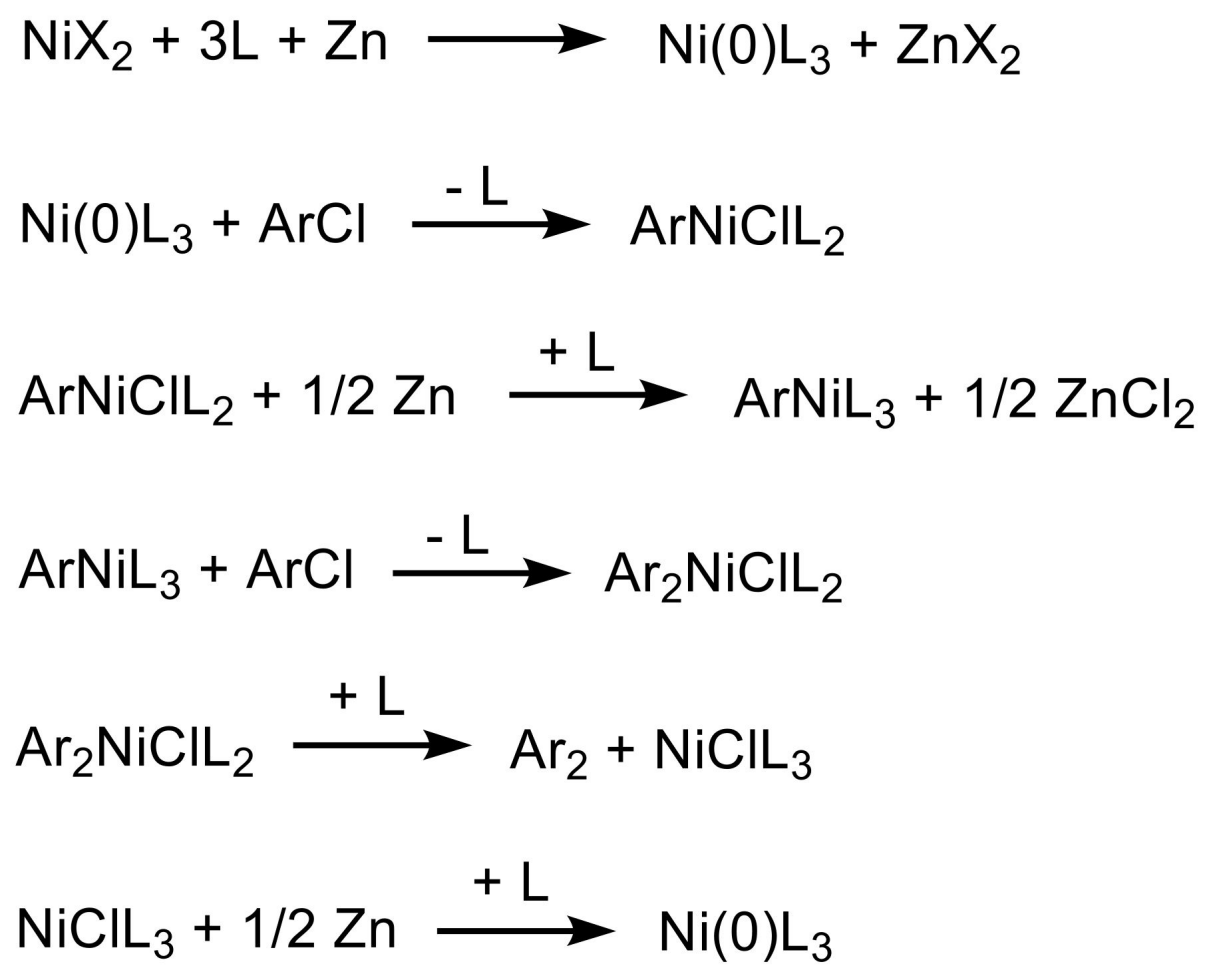
Mechanism of the nickel coupling reaction with NiX_2_ [43].

Ni(COD)_2_ is known to promote the carbon–carbon coupling reaction of aryl halides in the presence of BPY as a ligand in polar solvents [41]. As shown in Scheme 5, the oxidative addition of the aryl (Ar) halide to Ni(BPY)(COD) gives NiX(Ar)(BPY) in an early stage of the reaction. The NiX(Ar)(BPY) complexes then undergo a disproportionation reaction, followed by the formation of carbon–carbon aryl bonds. However, in the coupling reaction using NiX_2_ as the catalyst, the NiX_2_(BPY) complex is known to have significantly lower reactivity toward aryl halides; thus, this catalyst works well only for the most reactive aryl iodides [42]. Therefore, as introduced by Colon and Kelsey (Scheme 6) [43], PPh_3_ must be incorporated as a ligand to stabilize the catalytically active Ni(0) complexes, aryl nickel(II), and diaryl nickel intermediates. In this system, zinc metal is used to reduce Ni(II)(PPh_3_)_2_X_2_ in the presence of PPh_3_ to the catalytically active Ni(0)(PPh_3_)_3_ complex. An excess of PPh_3_ is required because of the side reaction between PPh_3_ and zinc halides to obtain zinc complexes. Therefore, a different reagent was tried for each model reaction depending on the type of Ni catalyst, as shown in Table 1.

**Figure 1:**
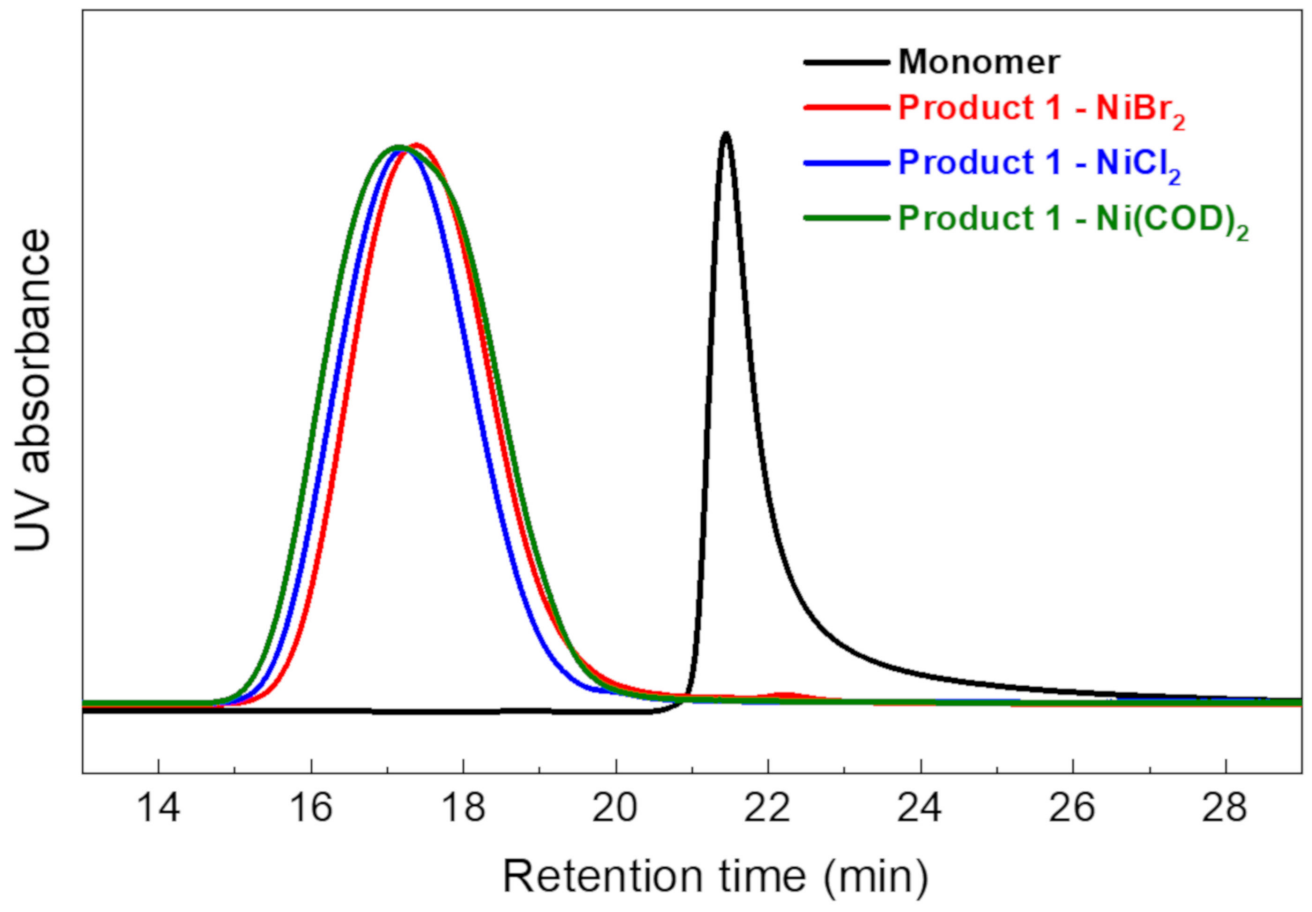
GPC traces of Product 1 with various nickel catalysts.

After the reaction, the molecular weights of Product 1 were estimated using GPC (Table 1), and the GPC traces are shown in Figure 1. All Ni(0) catalysts showed almost the same degree of polymerization, implying similar catalytic reactivity. The number average molecular weight according to GPC was 1.3 kDa for NiBr_2_*,* 1.5 kDa for NiCl_2_, and 1.4 kDa for Ni(COD)_2_. Their polydispersity index (PDI) values were also similar, ranging from 1.6 to 1.7, confirming that the catalytic ability of NiX_2_ is similar to that of Ni(COD)_2_ in the model reaction.

**Figure 2:**
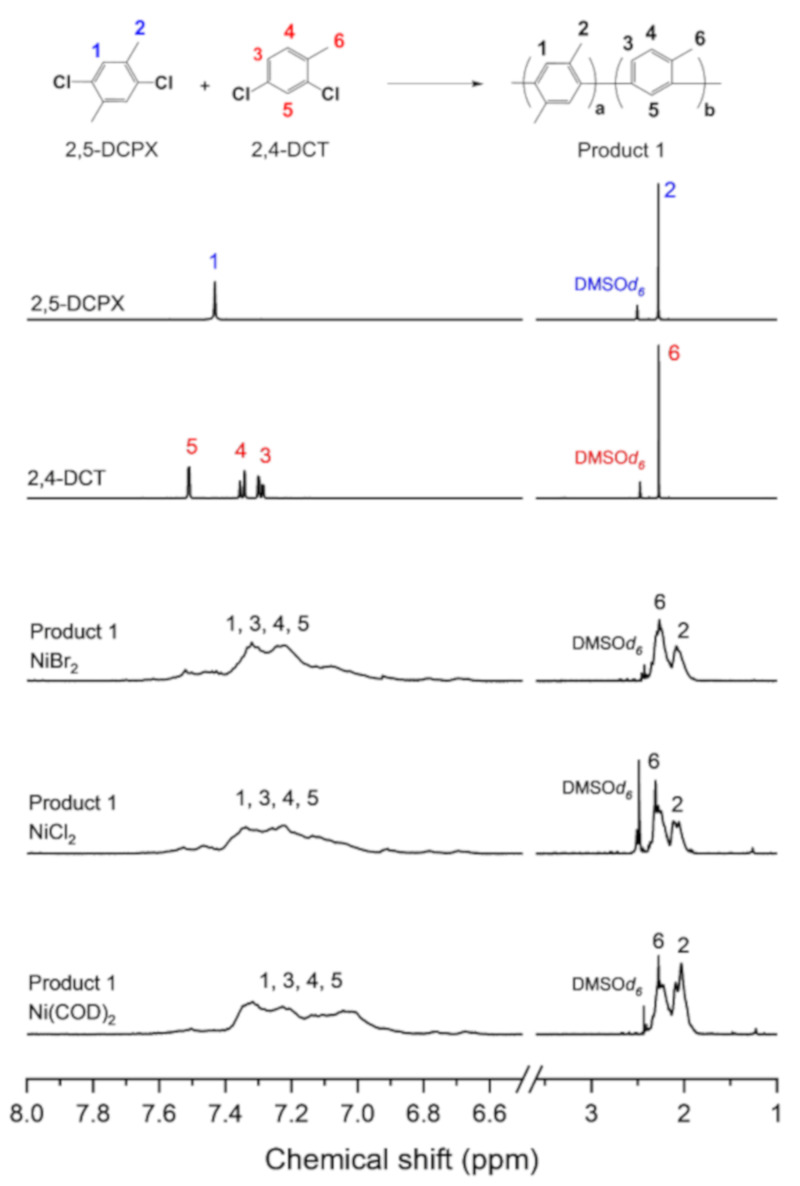
^1^H NMR spectra of 2,5-dichloro*-p-*xylene (2,5-DCPX), 2,4-dichlorotoluene (2,4-DCT), and Product 1 synthesized using NiBr_2_, NiCl_2_, or Ni(COD)_2_.

Figure 2 shows the ^1^H NMR spectra of 2,5-DCPX, 2,4-DCT, and Product 1, which was synthesized using NiBr_2_, NiCl_2_, or Ni(COD)_2_. Typical broad peaks for the phenylene structure (peaks 1, 3, 4, 5) are observed at 6.6–7.6 ppm for Product 1 in all three cases. Broad peaks 2 and 6 of the protons in the methyl group are also observed, indicating the successful reaction for all three catalysts.

One of the concerns in regards to the model reaction in Scheme 1 was the low molecular weight of the final polymer owing to the solubility of the growing phenylene polymer. Generally, the C–C coupling reaction does not require a stoichiometric balance for polymerization, which is known to be a common principle for polycondensation. In the case of a model step condensation reaction for aromatic polymers, oligomerization by stoichiometric imbalance can enable us to monitor the reactivity of catalysts or monomer alone because the designed low-molecular-weight aromatic oligomers do not have the solubility issue in the reaction solvent. However, the stoichiometric imbalance technique cannot be used for the C–C coupling reaction; thus, the degree of polymerization was affected not only by the solubility of the growing rigid phenylene polymer (or oligomer) but also the reactivity of the Ni catalyst. We have found a way to monitor the reactivity of the Ni catalyst during the model reaction because controlling the molecular weight was impossible during the C–C coupling reaction. Nevertheless, all Ni catalysts resulted in products with similar molecular weights, which means that similar reactivity was maintained if the growing polymer was soluble during the reaction.

**Figure 3:**
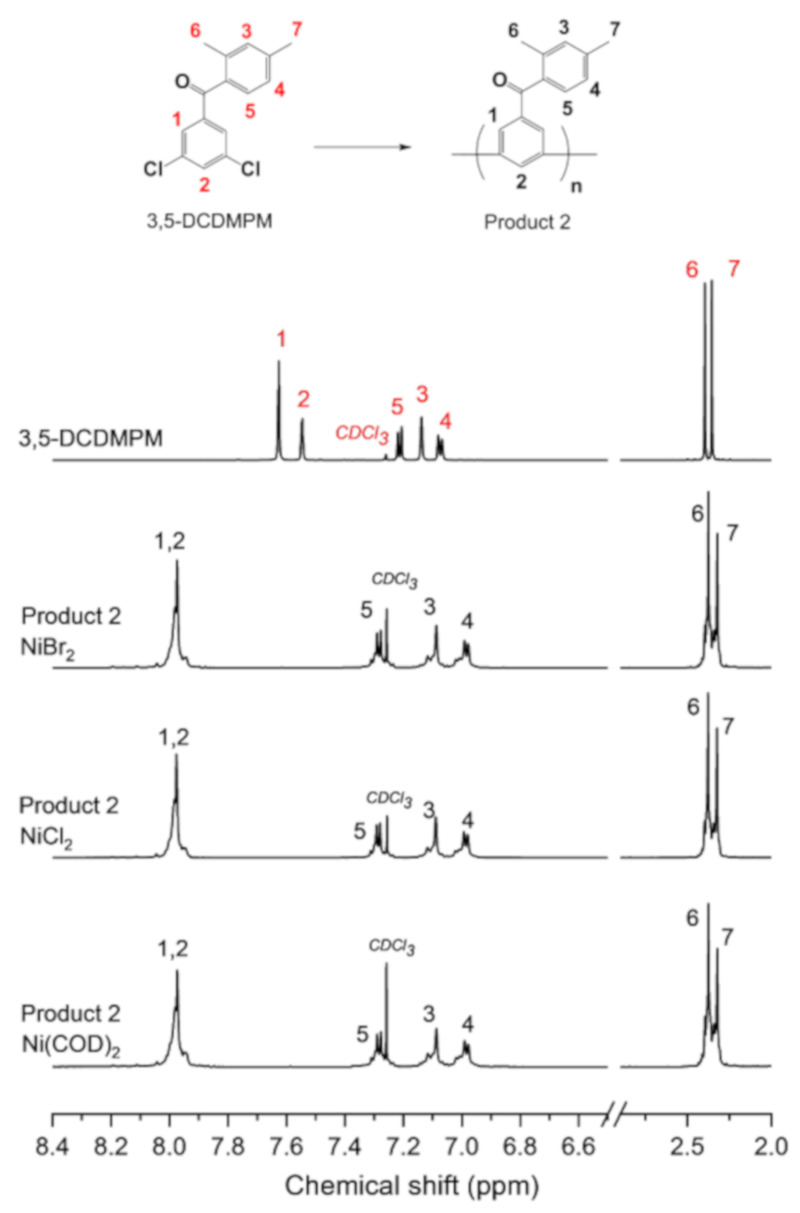
^1^H NMR spectra of (3,5-dichlorophenyl)-(2,4-dimethylphenyl)methanone (3,5-DCDMPM) and Product 2 synthesized using NiBr_2_, NiCl_2_, or Ni(COD)_2._

To validate this hypothesis, another model reaction was carried out using different monomer structures bearing benzophenone, which have frequently been used to fabricate p-phenyl electrolyte membranes [44,45]. Product 2 was synthesized from a 3,5-DCDMPM monomer, as shown in Scheme 2. The synthetic procedure of 3,5-DCDMPM and structure confirmation by ^1^H NMR are provided in Scheme S1 and Figure 3, respectively. The synthesis procedure of Product 2 was similar to that of Product 1.

**Figure 4:**
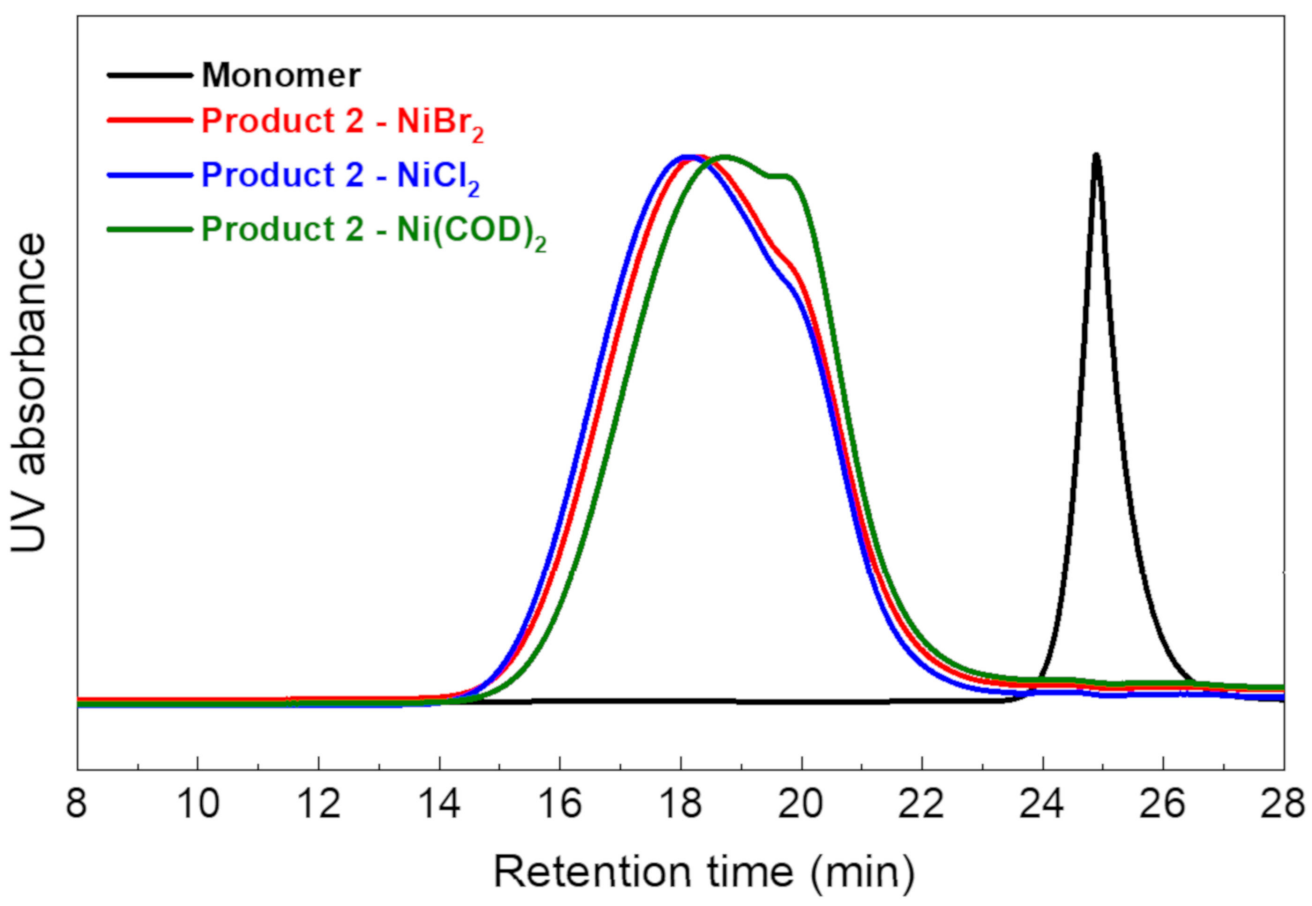
GPC traces of Product 2 with various Ni(0) catalysts.

Figure 4 shows the GPC traces of Product 2 synthesized using NiX_2_ and Ni(COD)_2_. The maximum retention time of the products is shorter than that of the monomer trace, indicating successful polymerization. The estimated molecular weights (Table 2) of the polymers synthesized using NiBr_2_ (*Mn* = 3.5 kDa, PDI = 1.80) and NiCl_2_ (*Mn* = 3.7 kDa, PDI = 1.81) are higher than that synthesized with Ni(COD)_2_ with *Mn* = 4.6 kDa, PDI = 2.20. This result confirms that NiX_2_ is a promising alternative to the expensive Ni(COD)_2_ for the coupling reaction. In addition, Product 2 generally has a higher molecular weight than Product 1. This result might be explained by the incorporation of the ketone group, which might increase the reactivity of the monomer toward the nickel catalysts [46]. The introduction of a larger side chain may also increase the flexibility of the phenylene polymer, hence increasing the maximum molecular weight.

Figure 3 shows the ^1^H NMR spectra of the 3,5-DCDMPM monomer and the corresponding Product 2 synthesized using NiBr_2_, NiCl_2_, or Ni(COD)_2_. Peaks 1 and 2 from 3,5-DCDMPM shift downfield in Product 2, and the remaining peaks 3–7 broaden, indicating the successful coupling reaction. Product 2 shows a similar pattern for all three catalysts, confirming the successful catalyzation by NiBr_2_ and NiCl_2_ without any side reactions.

### 3.2. Synthesis of Poly(Phenylene) from 3,5-DCBP Monomer and Chlorine-Terminated Hydrophobic Oligomer

**Figure 5:**
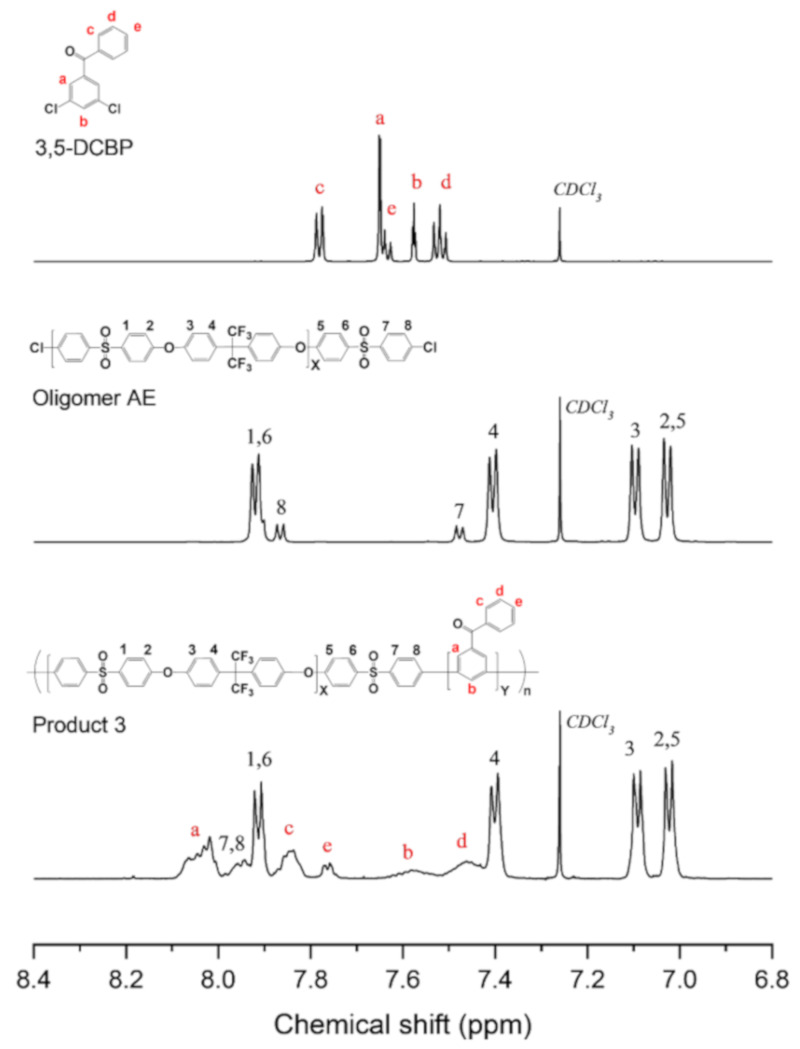
^1^H NMR of 3,5-DCBP, Oligomer AE, and Product 3 synthesized using Ni(COD)_2_.

Based on the model reactions of Products 1 and 2, we attempted another model reaction to polymerize a multi-block copolymer (Product 3) using the three Ni(0) catalysts. Although NiBr_2_ and NiCl_2_ showed similar results to Ni(COD)_2_ for synthesizing random poly(phenylene) from dichloro-monomers, the polymerization of the multi-block copolymer from a chlorine-terminated oligomer generally required a much higher degree of reactivity. Thus, we designed a relatively simple model multi-block copolymer that does not bear an ionic functional group. Although using a functionalized monomer is preferred to avoid the undesired halogenation process, as discussed in the introduction [20], we intended to minimize the effect of such a functional group on the reactivity of catalysts in the synthesis of Product 3. Functionalized monomers, such as those with amine or ammonium groups, have been reported to hinder the degree of polymerization [43]. The 3,5-DCBP monomer was selected as the hydrophilic block, and Oligomer AE was selected as the hydrophobic block in the model, as shown in Scheme 3. The preparation procedure for 3,5-DCBP is available in the Supporting Information as Scheme S2, and its chemical structure was confirmed by ^1^H NMR, as depicted in Figure 5. Meanwhile, the flexible Oligomer AE was introduced to make the polyphenylene structure of the multi-block backbone less rigid and to obtain a high molecular weight polymer. Oligomer AE was prepared via a nucleophilic aromatic substitution, as described in the Supporting Information (Scheme S3), and the structure was also confirmed by ^1^H NMR (Figure 5).

**Figure 6:**
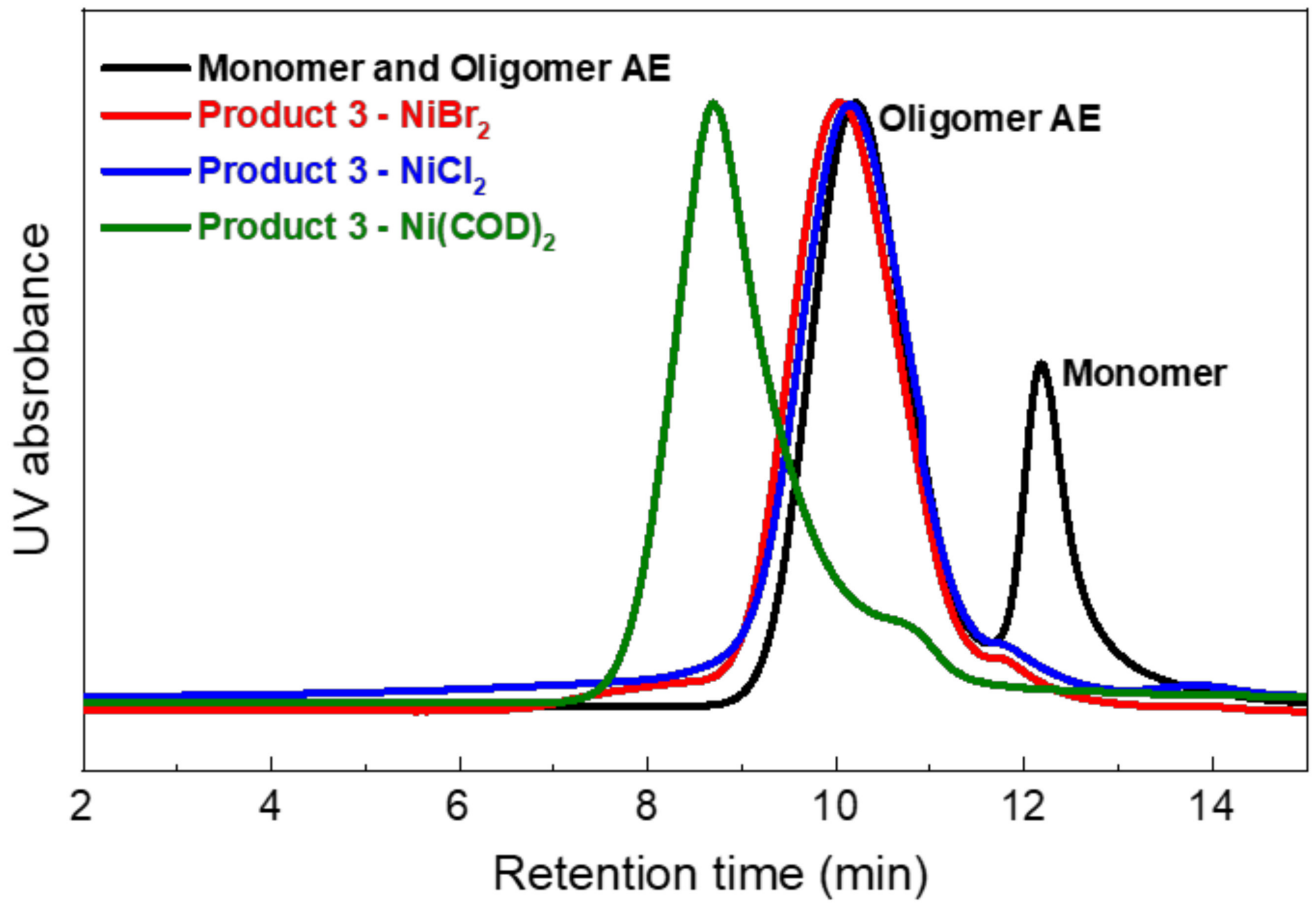
GPC traces of Product 3 synthesized using various nickel catalysts.

The nickel coupling reactions for Product 3 followed a mechanism similar to those for Products 1 and 2. The GPC traces of the starting monomer, Oligomer AE, and the polymer products are shown in Figure 6. The polymer synthesized using Ni(COD)_2_ showed a shorter retention time than Oligomer AE, suggesting the successful polymerization process. Figure 5 shows the ^1^H NMR spectra of Product 3, confirming the chemical structure of the product synthesized using Ni(COD)_2_. Peaks 1–6 from Oligomer AE remain intact, while peaks 7–8 of the end groups are shifted downfield. Peaks a–e from 3,5-DCBP are significantly shifted and broadened, typical of the poly(phenylene)s structure. The integration ratios of peaks a–e and peaks 1–6 show that the product has X5Y5, similar to the theoretical value.

In contrast, the GPC traces shift only slightly in the case of the NiBr_2_ and NiCl_2_ catalysts, implying unsuccessful reactions. The estimated molecular weights of the polymers are summarized in Table 3. Using Ni(COD)_2_ resulted in a high-molecular-weight copolymer with *M_w_* = 131.0 kDa and PDI = 5.6, which were significantly higher than those of NiBr_2_ (*M_w_* = 12.3 kDa and PDI = 2.2) and NiCl_2_ (*M_w_* = 14.2 kDa and PDI = 2.1). The two NiX_2_ catalyst systems that were used in the reaction, with phosphorous bidentate ligands, are suspected of having insufficient reactivity with the Oligomer AE. Meanwhile, Ni(COD)_2_ with the incorporation of BPY as the ligand had higher reactivity with the reactants than the NiX_2_ systems [41].

Another possible reason for the failure to synthesize Product 3 is the different reactivity of the monomers. Multi-block copolymerization started with a combination of Cl-terminated oligomers and monomers. The oligomers with a Cl^-^ end group would have much lower reactivity than the corresponding monomers owing to their lower mobility, which might hinder the accessibility of the Ni(0) catalyst, as shown in Scheme 4, resulting in an unsuccessful Ar–Ni coupling reaction. Moreover, sulfone (-SO_2_-) attached to the chlorobenzene end group could lower its electron density, which has been reported to affect the reactivity of the C–C coupling reaction [46]. In any case, the catalytic reactivity of NiX_2_ appears to be insufficient for multi-block copolymerization with oligomers.

### 3.3. Synthesis of Amine-Containing Poly(Phenylene) Block Copolymer with Ni(COD)_2_

**Figure 7:**
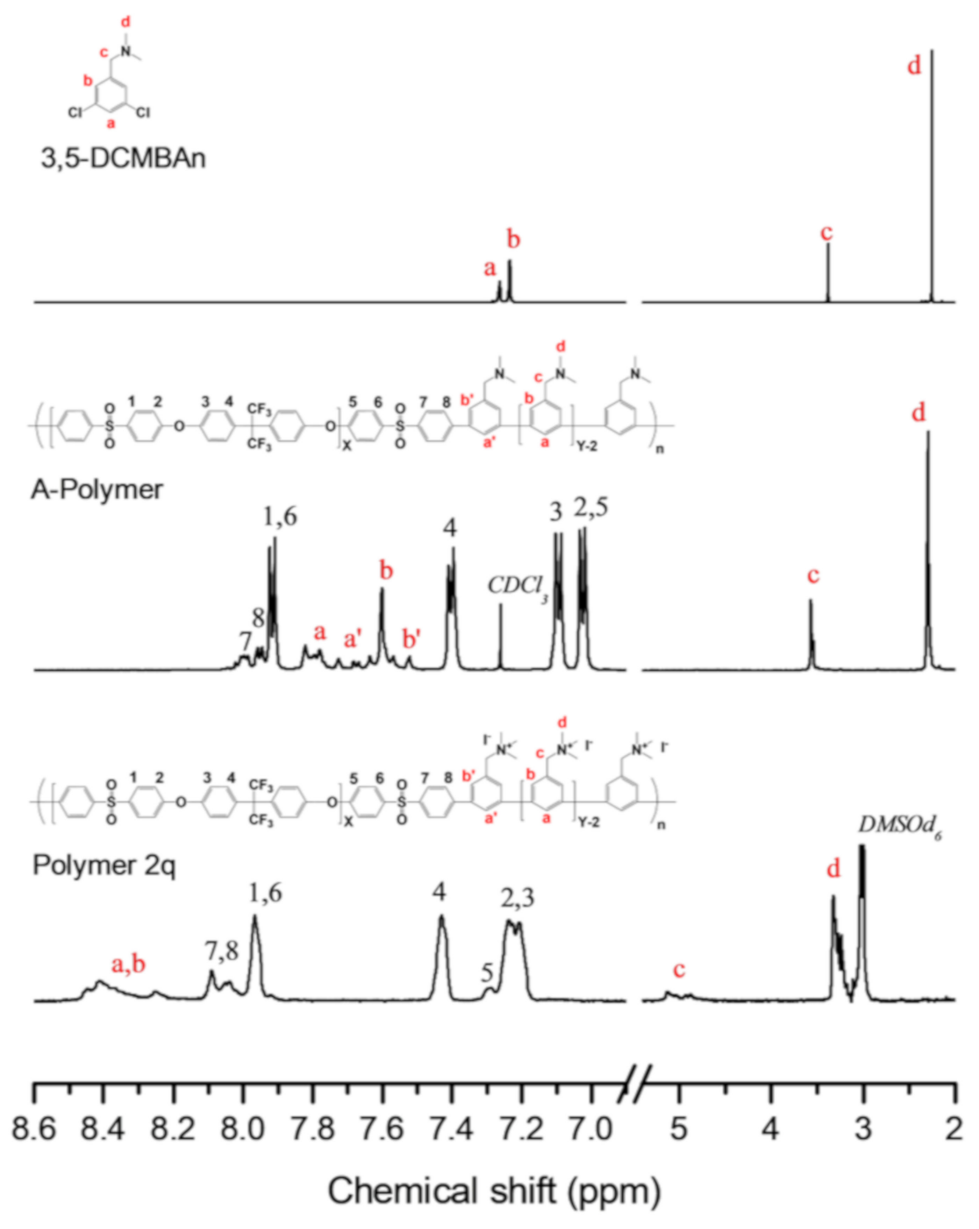
^1^H NMR spectra of 3,5-DCMBAn, A-Polymer, and Q-Polymer.

**Figure 8:**
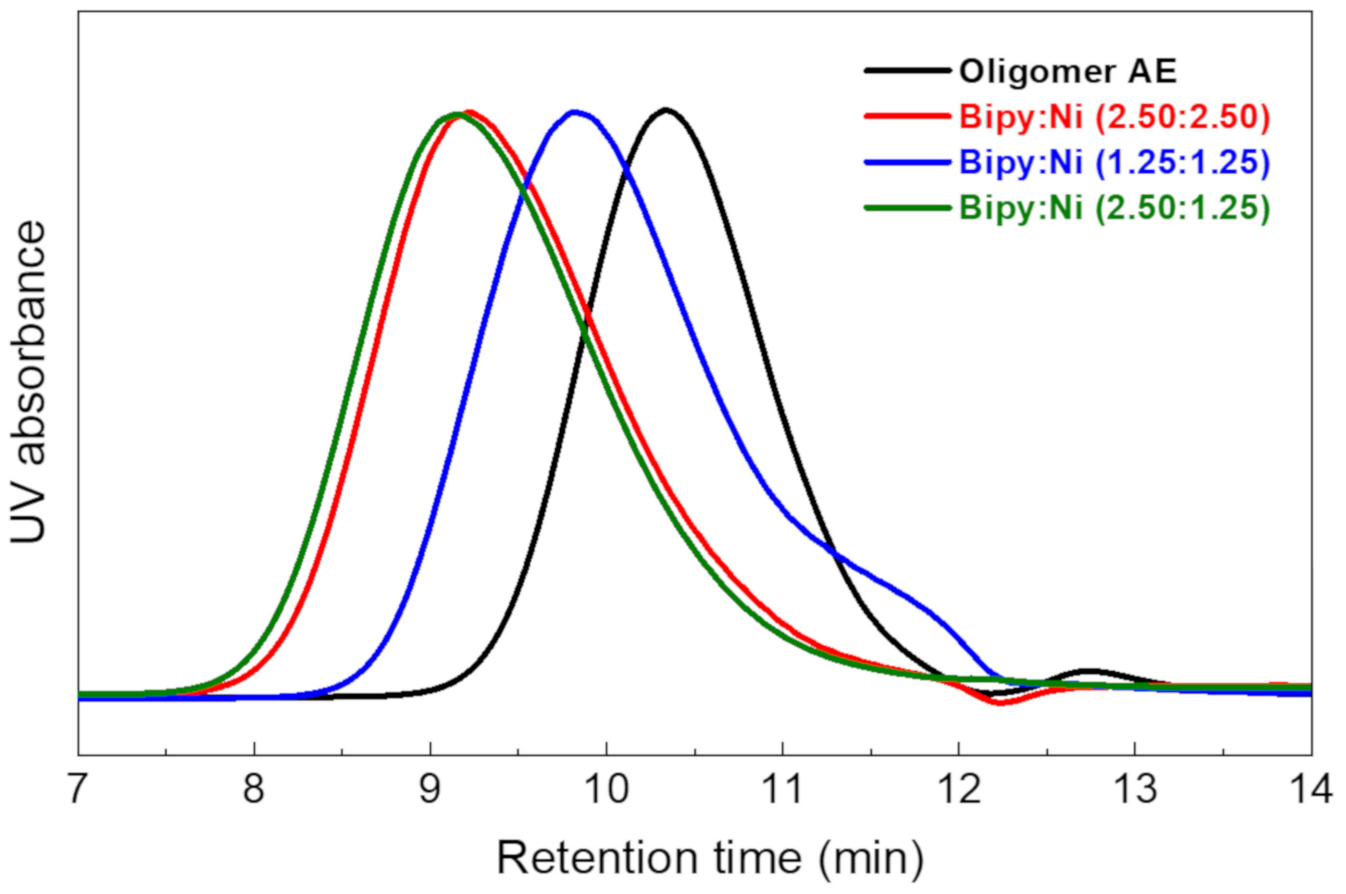
GPC traces of A-Polymer synthesis.

Based on the results of synthesizing Product 3, only Ni(COD)_2_ was selected for further study, and the research aim became to decrease the use of expensive Ni(COD)_2_ as much as possible to synthesize AEM multi-block copolymers. The amine-containing poly(phenylene) block copolymer (Polymer) was synthesized from Oligomer AE as the hydrophobic block component and 3,5-DCMBAn as the hydrophilic block component. The preaminated 3,5-DCMBAn was chosen to avoid the halogenation process, as mentioned in Section 1, and it was prepared by the reduction reaction of 3,5-dichloro-*N,N*, dimethybenzamide [47], which was synthesized from 3,5-DCBC via the Schotten–Baumann reaction [48]. The chemical structure of 3,5-DCMBAn was confirmed by ^1^H NMR, as shown in Figure 7. The multi-block copolymer was synthesized by using different stoichiometric ratios of Ni(COD)_2_ (Table 4). First, we used a stoichiometric ratio for the model reactions with 2.5 mol of excess Ni(COD)_2_ and BPY, similar to that for previously reported reactions [49,50]. The synthesized polymer showed a relatively high molecular weight (*M_w_* = 80.1 kDa) and low PDI (3.90) for phenylene polymers. Second, we decreased the amount of Ni(COD)_2_ and BPY to 1.25 mol in excess of the monomers and oligomers. This condition gave a low *M_w_* of 38.7 kDa. As an alternative approach, we increased the amount of BPY to 2.5 mol, while the amount of Ni(COD)_2_ was unchanged. This approach resulted in a high-molecular-weight polymer with *M_w_* = 93.9 kDa and PDI = 3.62. The molecular weight distribution and GPC traces are presented in Table 4 and Figure 8, respectively.

The chemical structure of the polymer was confirmed by ^1^H NMR, as shown in Figure 7. Peaks 1–8 represent the protons from the hydrophobic block. Peaks 1–6 are present, and peaks 7 and 8 are shifted, signifying the coupling reaction of the oligomer end group. Peaks a and b represent the protons from the 3,5-DCMBAn monomers, which shifted after polymerization. The ion exchange capacity (IEC) was estimated from the ratio of the integral area between the hydrophilic and hydrophobic block peaks. The polymer was estimated to have an IEC of 1.77 meq/g, similar to the theoretical value of 1.80 meq g^−1^ calculated from the feed ratio of Oligomer AE and the monomers.

Although the amount of Ni(COD)_2_ was decreased by 50%, the C–C coupling reaction was still successful. To the best of our knowledge, this amount is the lowest ever reported for p-phenylene polymers. The role of BPY seems to be very distinct based on the results of polymerization because the excess feed of BPY dramatically increased the degree of polymerization. Although the reason for the high molecular weight remains unclear, we can postulate the role of BPY, as shown in the mechanism in Scheme 6. The excess amount of BPY seems to function as a ligand to help generate a Ni(BPY)(COD) intermediate during polymerization. Specifically, the successful formation of a Ni(BPY)(COD) intermediate was favored over NiX(Ar)(BPY), which can induce a disproportionation reaction and the formation of Ar–Ar (Scheme 6).

### 3.4. Membrane Preparation, Quaternization, and Characterization

The amine-containing poly(phenylene) block copolymer was soluble in organic solvents such as chloroform, NMP, and DMAc. A membrane with a thickness of 50 ± 3 µm was obtained by solution casting followed by quaternization to provide anion-exchange groups. The quaternized membrane was soluble in DMSO-*d_6_*, but it was not soluble in the chloroform, DMAc, or NMP, which implied a successful quaternization process. The ^1^H NMR in Figure 7 confirms the full conversion of benzylamine to benzyl ammonium by comparing the integration value of protons c and d. The IEC value (1.77 meq g⁻^1^) was also confirmed by calculating the integration ratio between proton peaks of the hydrophilic block (a–d) and those of the hydrophobic block (1–8) and comparing the value to the aminated precursor polymers.

**Figure 9:**
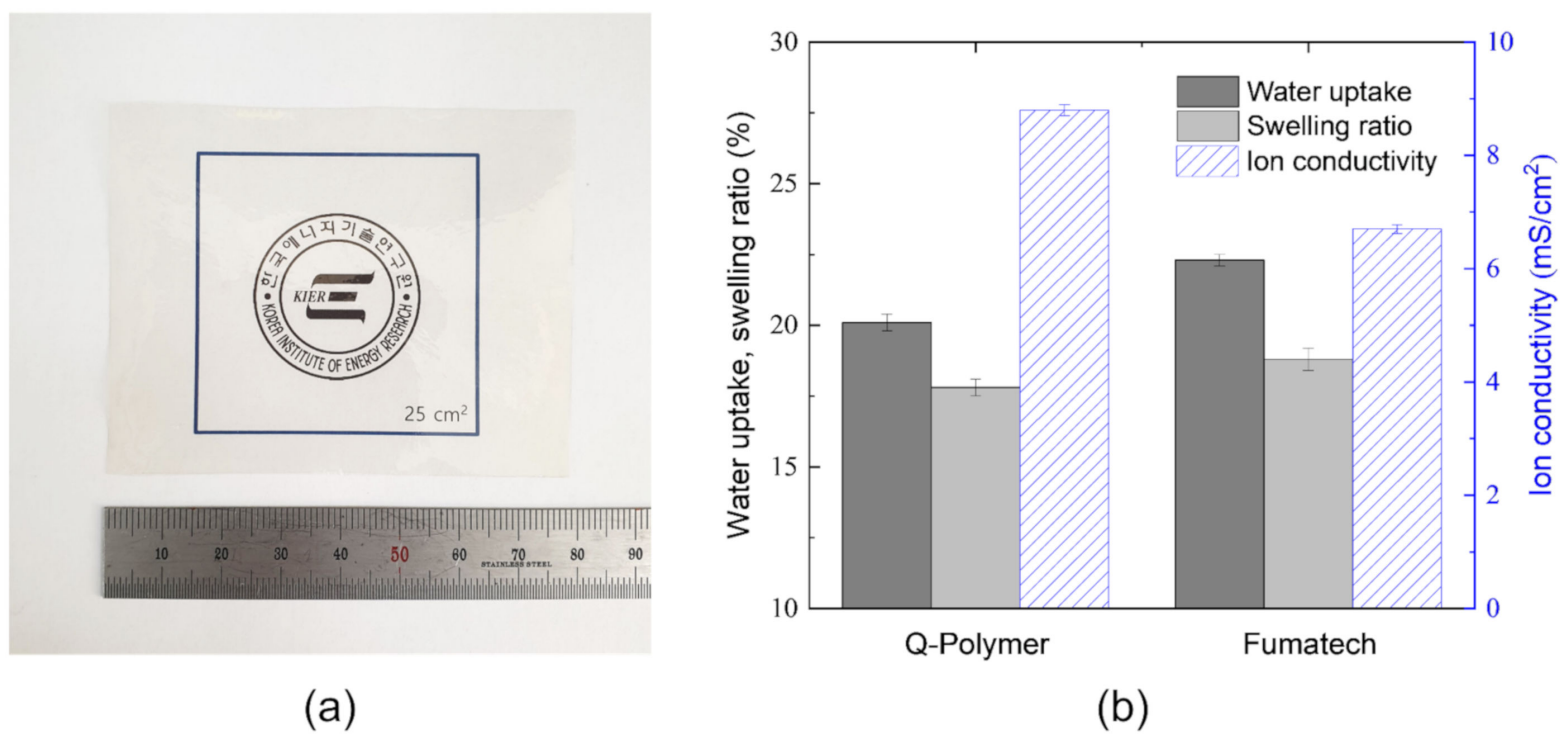
(**a**) Photograph of the Q-Polymer membrane (Cl^−^ form) and (**b**) comparison of the water uptake, swelling ratio, and ion conductivity of Q-Polymer and Fumatech (FAA-3-50) membranes measured in deionized water (DIW) at 25 °C.

The water uptake, swelling volume ratio, and ion conductivity of the quaternized poly(phenylene) multi-block copolymer (Q-Polymer) membrane were measured at 25 °C in DIW and compared with those of the commercially available Fumatech membrane (FAA-3-50, 2.0 meq g^−1^). The anion of the quaternized membrane was converted into the chloride form for a better comparison and to avoid carbon dioxide contamination. The membrane in the chloride form was bendable, ductile, transparent, and colorless (Figure 9a), and its membrane performance parameters are summarized in Figure 9b. The Q-Polymer has a lower water uptake (20.1%) and volume swelling ratio (17.8%) than the Fumatech membrane (22.3% and 18.8%, respectively), which might be due to the lower IEC of the block copolymer. On the other hand, the Q-Polymer has higher chloride conductivity (8.8 mS cm^−1^) than the Fumatech membrane (6.7 mS cm^−1^). Further, to evaluate the overall performance of the membranes with similar IEC values, a characteristic factor (Φ) was calculated as the ratio of the chloride conductivity to the water volume fraction [51]. The Q-Polymer exhibits a higher Φ value (0.031) than the Fumatech membrane (0.022), suggesting more efficient ion transport, despite its lower water content.

**Figure 10:**
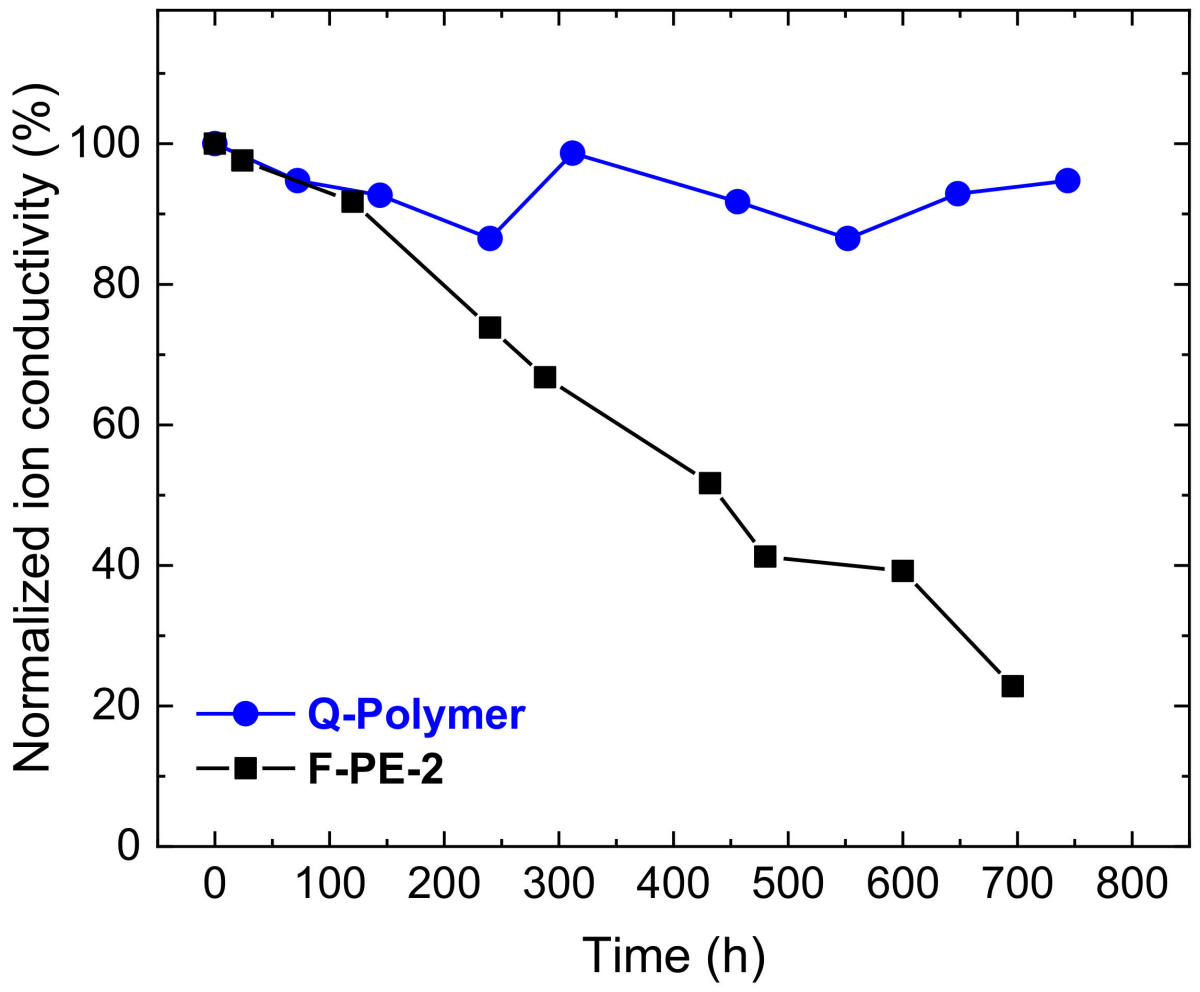
Normalized hydroxide ion conductivity of the Q-polymer membrane during stability testing in a 1 M NaOH solution at 25 °C for 700 h, compared with the results of a poly(arylene ether) based membrane, F-PE-2. Adapted with permission from reference [19]. Copyright 2017 Elsevier.

The long-term chemical stability of the Q-Polymer was tested under strong alkaline conditions in a similar manner to our previous research to compare its stability with that of the poly(arylene ether) block copolymer membrane (F-PE-2) in that study [19]. Figure 10 shows their hydroxide ion conductivities normalized to their initial values. The Q-Polymer membrane was significantly more stable, retaining 95% of its conductivity after 700 h, in contrast with F-PE-2, which retained only 24%. This result suggests that introducing the poly(phenylene) structure into the hydrophilic block could improve the chemical stability of the membrane. The degradation of the main chain was probably suppressed by the higher bond dissociation energy of C–C bonds than that of C–O bonds in the ether substituent [22]. Thus, multi-block poly(phenylene) was successfully synthesized with a lower amount of Ni(COD)_2_ to obtain a promising membrane for applications in AEMs.

## 4. Conclusions

Several approaches for synthesizing a poly(phenylene)-based block copolymer were investigated with NiBr_2_, NiCl_2_, and Ni(COD)_2_ catalysts for the carbon–carbon coupling reaction. The low-cost NiBr_2_ and NiCl_2_ catalysts showed promising results for the first two model reactions, resulting in a polymer with a molecular weight similar to that achieved with Ni(COD)_2_. However, NiBr_2_ and NiCl_2_ catalysts did not successfully catalyze the model multi-block reaction using a chlorine-terminated oligomer and 3,5-DCBP to synthesize a high-molecular-weight polymer. Thus, another approach was explored to decrease the amount of Ni(COD)_2_ used to synthesize an amine-containing poly(phenylene) multi-block copolymer. As a result, a high-molecular-weight polymer was obtained by using 1.25 mol of Ni(COD)_2_ in excess of the reactant −50% less than that commonly used in a similar kind of synthesis. The membrane showed a relatively lower water uptake with higher anion conductivity than the commercial Fumatech membrane in DIW at 25 °C. In addition, introducing the poly(phenylene) backbone in an hydrophilic block could enhance the membrane’s chemical stability. Thus, this membrane is a potential alternative to commercial Fumatech membranes, and research on poly(phenylene) multi-block copolymers will be continued in the future. To that end, the fuel cell performance of the polymer and its derivatives will be explored in detail.

## Figures and Tables

**Table 1:** Product 1 syntheses and molecular weights.

Sample	Reactants (mmol)	Catalyst (mmol)	Zn (mmol)	BPY (mmol)	PPh_3_ (mmol)	M_n_^a^ (kDa)	M_w_^a^ (kDa)	PDI
NiBr_2_	6.4	0.5	27.5	-	3.2	1.3	2.0	1.6
NiCl_2_	6.4	0.5	27.5	-	3.2	1.5	2.3	1.6
Ni(COD)_2_	2.9	7.2	-	7.2	-	1.4	2.4	1.7

^a^ Estimated by GPC analyses (calibrated with polystyrene standards).

**Table 2:** Product 2 syntheses and molecular weights.

Sample	Reactants (mmol)	Catalyst (mmol)	Zn (mmol)	BPY (mmol)	PPh_3_ (mmol)	M_n_^a^ (kDa)	M_w_^a^ (kDa)	PDI
NiBr_2_	0.65	0.05	2.75	-	0.32	3.5	6.2	1.8
NiCl_2_	0.65	0.05	2.75	-	0.32	3.7	6.7	1.8
Ni(COD)_2_	0.65	1.63	-	1.63	-	2.1	4.6	2.2

^a^ Estimated by GPC analyses (calibrated with polystyrene standards).

**Table 3:** Product 3 syntheses and molecular weights.

Sample	Reactants (mmol)	Catalyst (mmol)	Zn (mmol)	BPY (mmol)	PPh_3_ (mmol)	XY ^a^	M_n_^b^ (kDa)	M_w_^b^ (kDa)	PDI
NiCl_2_	2.9	0.2	12.2	-	1.4	X5Y5	5.6	12.3	2.2
NiBr_2_	2.9	0.2	12.2	-	1.4	X5Y5	6.7	14.3	2.1
Ni(COD)_2_	2.9	7.3	-	7.3	-	X5Y5	23.4	131.0	5.6

^a^ Calculated from the feeding amount ratio. ^b^ Estimated by GPC analyses (calibrated with polystyrene standards).

**Table 4:** Catalyst ratio and molecular weight distribution of the polymer.

Reactants/BPY/Ni(COD)_2_ Ratio	M_n_^a^ (kDa)	M_w_^a^ (kDa)	PDI
1:2.5:2.5	20.5	80.1	3.9
1:1.25:1.25	2.9	38.7	13.1
1:2.5:1.25	25.9	93.9	3.6

^a^ Estimated by GPC analyses (calibrated with polystyrene standards).

## Supplementary Materials

The following are available online at https://www.mdpi.com/2073-4360/12/7/1614/s1, Synthesis of (3,5-dichlorophenyl)-(2,4-dimethylphenyl)methanone, Synthesis of 3,5-dichlorobenzophenone, Synthesis of chlorine-terminated telechelic hydrophobic oligomers, Scheme S1. Synthesis of 3,5-DCDMPM; Scheme S2. Synthesis of 3,5-DCBP; Scheme S3. Synthesis of Oligomer AE.
