# Supplementary material for: Synthetic Approaches for Poly(Phenylene) Block Copolymers via Nickel Coupling Reaction for Fuel Cell Applications
Source: Polymers (Basel). 2020 Jul 20;12(7):1614. doi: 10.3390/polym12071614 (PMC7407201; doi:10.3390/polym12071614)
Supplement: Supplementary file 1 [file polymers-12-01614-s001.pdf]

Supporting Information

Synthetic Approaches for Poly(Phenylene) Block Copolymers via Nickel Coupling Reaction for Fuel Cell Applications

Adam F. Nugraha [1,2,†], Songmi Kim [1,3,†], Farid Wijaya [1,2], Byungchan Bae [1,2] and Dongwon Shin [1,*]

1 Fuel Cell Laboratory, Korea Institute of Energy Research, Daejeon 34129, Korea; adamfn@kier.re.kr (A.F.N.); songmk307@kier.re.kr (S.K.); faridwijaya@ust.ac.kr (F.W.); bcbae@kier.re.kr (B.B.)

2 Department of Renewable Energy Engineering, University of Science and Technology (UST), Daejeon 34113, Korea

3 Department of Chemical Engineering, Yonsei University, Seoul 03722, Korea

* Correspondence: dwshin@kier.re.kr; Tel.: +82-42-860-3019; Fax: +82-42-860-3104

† Both authors contributed equally to this manuscript.

Received: 25 June 2020; Accepted: 18 July 2020; Published: date

1. Synthesis of (3,5-dichlorophenyl)-(2,4-dimethylphenyl)methanone

(3,5-Dichlorophenyl)-(2,4-dimethylphenyl)methanone (3,5-DCDMPM) was synthesized from 3,5-dichlorobenzoyl chloride (3,5-DCBC) and *m*-xylene via a Friedel–Crafts reaction, as shown in **Error! Reference source not found.**1. The typical procedure is as follows: *m*-xylene (246.3 mmol, 30 mL) and $AlCl_3$ (37.2 mmol, 5.0 g) were stirred in a 100 mL round-bottomed four-necked flask equipped with a magnetic stirrer, a condenser, a dropping funnel, a nitrogen inlet/outlet, and an ice bath. 3.5-DCBC (28.6 mmol, 6.0 g) was added dropwise, the ice bath was removed, and the temperature was increased to 60 °C slowly and maintained for 6 h. After the reaction, the organic layer was extracted with toluene, followed by successive washing with water, saturated sodium bicarbonate aqueous solution, and deionized water. The obtained white precipitate after evaporation was recrystallized in an EtOH/DIW (8:2) mixture. The white crystals were filtered and dried in a vacuum oven at 50 °C overnight (yield 92.3%).

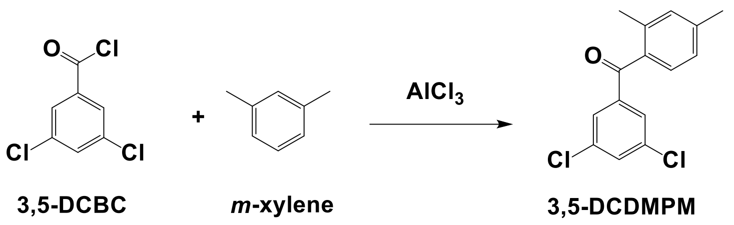

Scheme S1. Synthesis of (3,5-Dichlorophenyl)-(2,4-dimethylphenyl)methanone (3,5-DCDMPM).

2. Synthesis of 3,5-dichlorobenzophenone

A typical procedure for 3.5-DCBP is as follows (Scheme S2). A 100 mL round-bottomed four-necked flask equipped with a condenser, a magnetic stirrer, a dropping funnel, and a nitrogen inlet/outlet was charged with benzene (245.8 mmol, 25 mL) and $AlCl_3$ (37.24 mmol, 5.0 g) in an ice bath. 3.5-DCBC (28.6 mmol, 6.0 g) was added dropwise, the temperature was increased to 60 °C slowly, and the reaction was maintained for 5 h. The mixture was quenched, acidified, and extracted with toluene. The organic layer was washed with water, saturated sodium bicarbonate aqueous solution, and deionized water, successively. The organic layer was collected and evaporated using a rotary

evaporator. The obtained precipitate was recrystallized in an EtOH/DIW (8:2) mixture. The obtained white crystal was filtered and dried in a vacuum oven at 50 °C overnight (yield 90.0%).

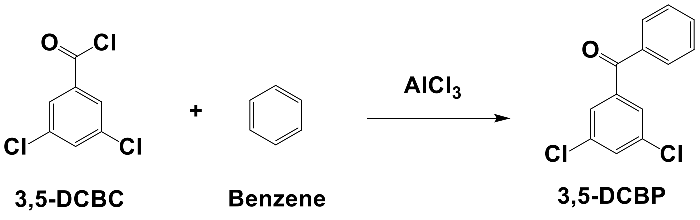

Scheme S2. Synthesis of 3,5-DCBP.

3. Synthesis of chlorine-terminated telechelic hydrophobic oligomers

The chlorine-terminated telechelic hydrophobic oligomer (Oligomer AE) was synthesized via nucleophilic aromatic substitution, as shown in Scheme S3. A typical procedure is as follows: (X = 5; X is the repeating unit of the hydrophobic oligomer), 2,2-bis(4-hydroxyphenyl)hexafluoropropane (5.0 g, 14.9 mmol), and 4,4'-dichlorodiphenyl sulfone (5.1 g, 17.8 mmol) were dissolved in NMP (40 mL) of a 100-mL round-bottom four-neck flask equipped with a magnetic stirrer and condenser under argon purging conditions. The temperature was raised to 140 °C, and potassium carbonate (2.5 g, 17.9 mmol) was added. The reaction was carried out for 6 h, and its progress was monitored by gel permeation chromatography (GPC). The obtained mixture was diluted and poured into deionized water. The solid was washed with pure methanol several times and was dried under vacuum at 80 °C overnight to produce Oligomer AE as a white powder (7.4 g, 92.2% yield).

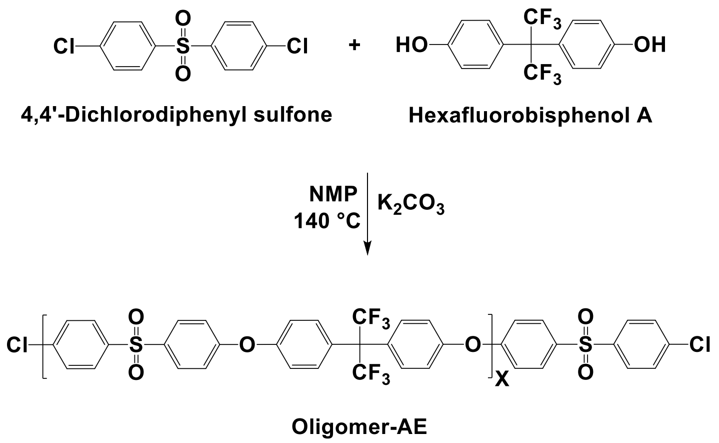

Scheme S3. Synthesis of aryl ether oligomer (Oligomer AE).
